# Appendiceal Mucinous Adenocarcinoma Presenting as Flank and Thigh Abscess: An Unusual Extra-abdominal Manifestation in a Rural Setting

**DOI:** 10.7759/cureus.105254

**Published:** 2026-03-15

**Authors:** Zachary Pluim, Sena Park, Shaheen Hasmat

**Affiliations:** 1 General Surgery, Dubbo Base Hospital, Dubbo, AUS

**Keywords:** appendiceal adenocarcinoma, mucinous cystic neoplasm, perforated appendicitis, retroperitoneal abscess, thigh abscess

## Abstract

Appendiceal malignancies are rare clinical entities, most commonly diagnosed incidentally following appendicectomy. When symptomatic, these tumors frequently mimic acute appendicitis, which may obscure the underlying diagnosis. Presentation with extra-abdominal symptoms, including skin and soft tissue infection, is exceptionally rare. We report a case of a 63-year-old male presenting to a rural hospital with sepsis and a right flank abscess. CT demonstrated a large retroperitoneal collection extending from the right paracolic gutter into the flank and groin, with an associated appendicolith. In the absence of interventional radiology, surgical incision and drainage were undertaken. Despite multiple surgical drainages, recurrence of collections necessitated laparoscopic right hemicolectomy. Initial histopathology revealed perforated appendicitis with a tubulovillous adenoma demonstrating high-grade dysplasia, without definitive invasive carcinoma. Despite apparent source control, the patient re-presented two months later with a larger collection, now extending into the right thigh. Repeat debridement and biopsy of the flank and right thigh abscess tissue confirmed a diagnosis of mucinous adenocarcinoma of appendiceal origin. Immunohistochemistry demonstrated intact mismatch repair protein expression, and molecular testing detected a BRAF mutation. The patient elected for palliative management.

This case highlights a rare presentation of appendiceal mucinous adenocarcinoma mimicking perforated appendicitis with flank and thigh abscess formation. Retroperitoneal tracking along fascial planes may allow for extra-abdominal disease manifestations. Appendiceal mucinous adenocarcinoma has been associated with high rates of perforation, and underlying malignancy should be considered in adult patients presenting with perforated appendicitis, especially in the context of persistent or recurrent abscess formation.

## Introduction

Appendiceal malignancy represents a rare clinical entity, most commonly diagnosed incidentally on histopathology specimens following appendicectomy [1]. These cancers can be broadly classified as epithelial or non-epithelial in origin, with mucinous adenocarcinoma representing approximately a third of primary appendiceal malignancies [2].

When symptomatic, presentation of appendiceal tumors frequently mimics acute appendicitis, including right lower quadrant abdominal pain, with perforation of the appendix not uncommon [3,4]. Diagnosis can pose a challenge, with 32-58% of patients with appendiceal neoplasms receiving a preoperative diagnosis of acute appendicitis [4,5]. The risk of associated neoplasm significantly increases in the presence of perforation or abscess formation [5]. Presentation of appendiceal malignancy as a soft tissue infection or abscess is exceedingly rare, with few cases describing abscess formation within the abdominal wall, flank, thigh, and groin [6-10]. These atypical presentations pose a diagnostic challenge and may delay definitive management. Management of recurrent abscess formation and complex intra-abdominal sepsis related to malignancy is particularly challenging in resource-limited rural settings.

## Case presentation

A 63-year-old male presented to a rural New South Wales hospital with generalized decline and decreased oral intake. He described mild associated abdominal pain, primarily within the right lower quadrant, for the past four to six weeks. He had been unwell for several months, with an acute decline in function and weight loss, with markedly limited mobility. The patient reported a history of rheumatic fever, but otherwise no other significant medical conditions. He denied taking any regular medications.

On examination, the patient appeared cachectic from the end of the bed. His abdomen was soft and non-distended. Within the department, the patient was febrile (38.9°C) and tachycardic (heart rate (HR) 124). Biochemistry on presentation revealed elevated inflammatory markers, including C-reactive protein (CRP) and white cell count (WCC) (Table 1). Renal function was normal (Table 1). Albumin was low, and the patient was mildly anemic (Table 1). Intravenous ceftriaxone and gentamicin were commenced for sepsis of unknown origin. He was subsequently transferred from a rural peripheral site to a regional hospital with imaging and surgical capabilities for further assessment.

**Table 1:** Patient laboratory values on presentation. The values demonstrate elevated inflammatory markers, mild anemia, hypoalbuminemia, and normal renal function. WCC: white cell count; CRP: C-reactive protein

Parameter	Result	Reference Range
WCC	11.5 × 10^9^/L (High)	4.0 - 11.0 × 10^9^/L
CRP	202 mg/L (High)	<5 mg/L
Hemoglobin	126 g/L (Low)	130 - 170 g/L
Albumin	19 g/L (Low)	30 - 44 g/L
Creatinine	39 umol/L (Low)	60 - 100 umol/L

**Figure 1:**
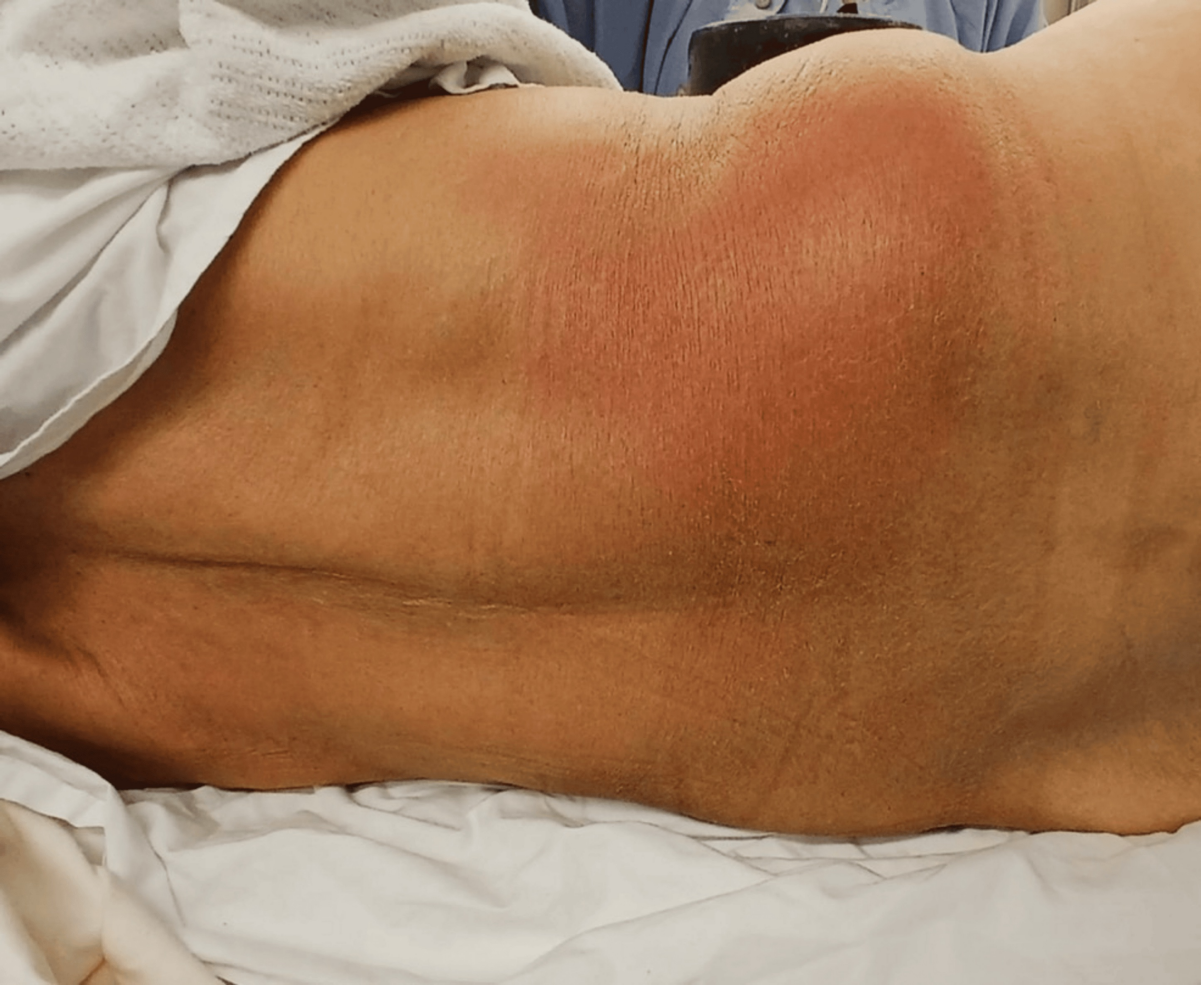
Clinical photograph demonstrating right flank abscess with overlying erythema.

Following transfer, re-examination revealed cellulitis, fluctuance, and tenderness overlying the right flank, with associated worsening tachycardia and hypotension concerning for sepsis (Figure 1).

**Figure 2:**
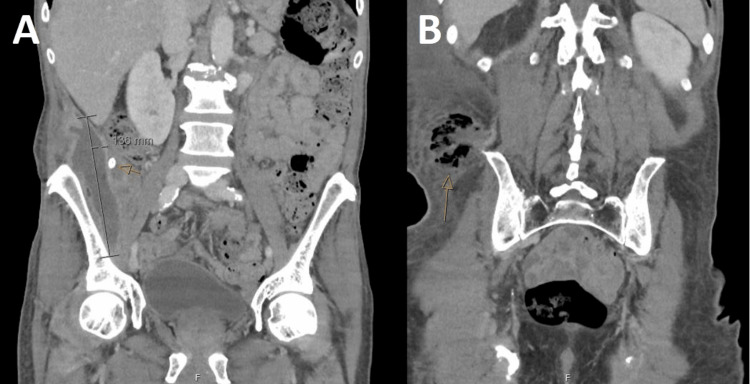
Coronal portal venous CT of the abdomen and pelvis. Demonstrating a large abscess (A) with free-floating calcification in keeping with fecalith (arrow) with extension to the right flank (B).

The patient was commenced on intravenous amoxicillin-clavulanate 2.2 g three times daily. A CT of the chest, abdomen, and pelvis was undertaken, revealing a large right paracolic gutter collection extending 13.5 cm craniocaudally, and anteriorly extending adjacent to the right psoas muscle to the right groin lying anteriorly to the hip joint (Figure 2). At points, this collection extended to the anterior abdominal wall. Imaging revealed an associated 11 mm calcification likely representing a fecalith with surrounding phlegmon within the right lower quadrant. These changes were deemed consistent with a perforated appendicitis with abscess formation.

In the context of sepsis and the unavailability of interventional radiology to facilitate radiologically guided drainage of the abscess, a decision was made to proceed with urgent incision and drainage of this flank collection. Preoperative bedside ultrasound was undertaken for localization, with a gas-containing right flank abscess identified. An incision was made overlying this area, in which a large abscess cavity containing 120 mL of frank pus was washed out and sent for microbiology, culture, and sensitivities, and a drainage tube was left.

**Figure 3:**
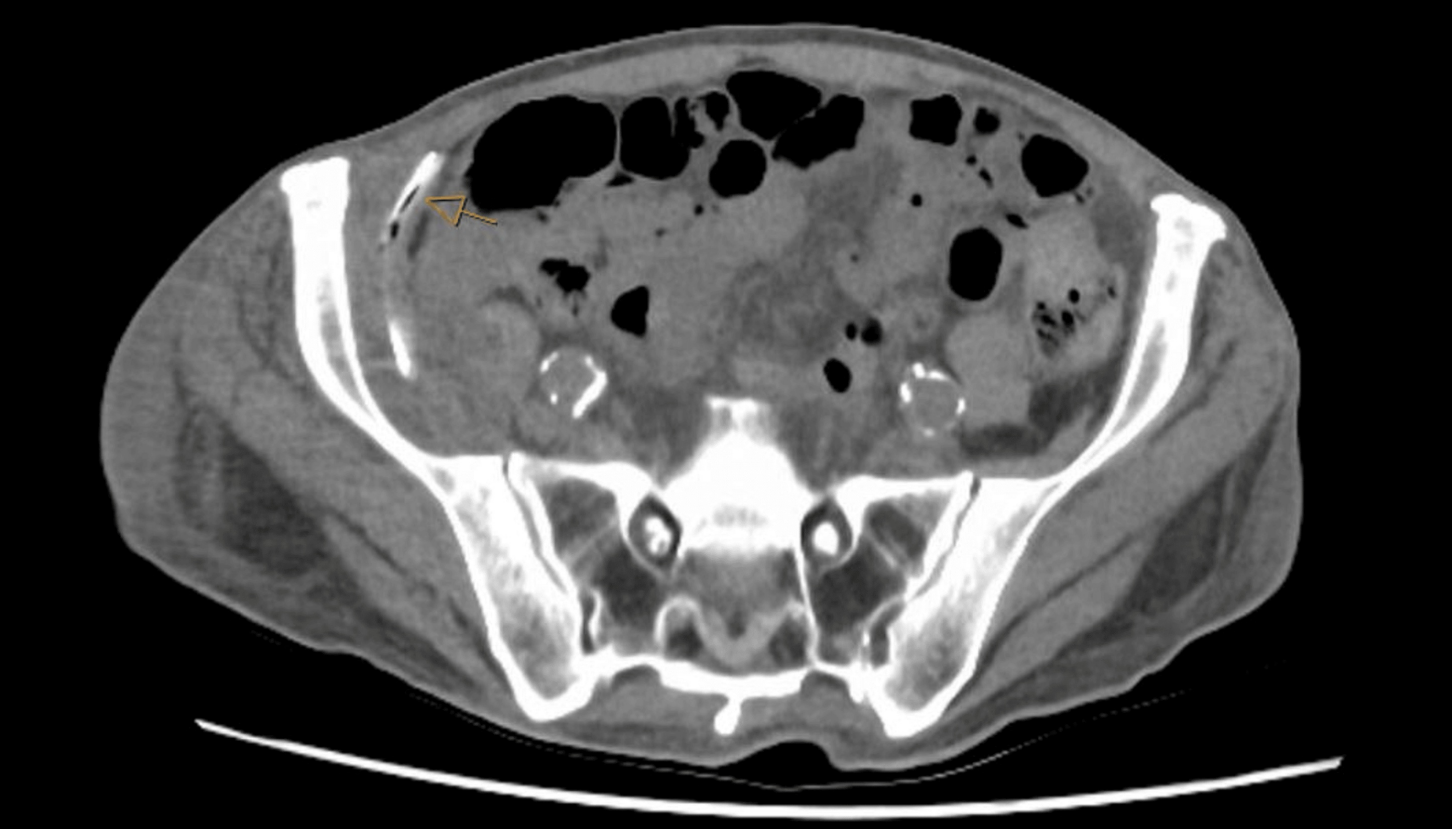
Axial portal venous CT of the abdomen and pelvis. Demonstrating a drain (arrow) extending into the retroperitoneal collection.

A repeat CT scan was undertaken on day one postoperatively, demonstrating an appropriately positioned drain around the iliac wing, extending to the deep aspect of this collection (Figure 3). The superficial aspect of this collection appeared adjacent to the drainage tubing.

The patient was managed with ongoing intravenous antibiotics, amoxicillin-clavulanate 2.2 g three times daily following his procedure, with ongoing drain output of 100-300 mL of frank pus daily. Repeat imaging was undertaken on the fourth day postoperatively, which revealed a significant improvement in the deep retroperitoneal component of the abscess; however, the superficial collection remained relatively unchanged. A decision was made to proceed with further incision and drainage the following day. An approximately 10 x 8 cm residual superficial collection was identified containing copious frank pus, with two drains inserted within this collection.

Drainage from the deep collection continued to decrease over the following three days postoperatively, with normalization of hemodynamic status and inflammatory markers. A decision was made for discharge with outpatient drain management and planning for further operative management of perforated appendicitis, including preoperative optimization of nutrition. He was discharged on oral amoxicillin-clavulanate 875 mg/125 mg twice daily for seven days while awaiting further review.

**Figure 4:**
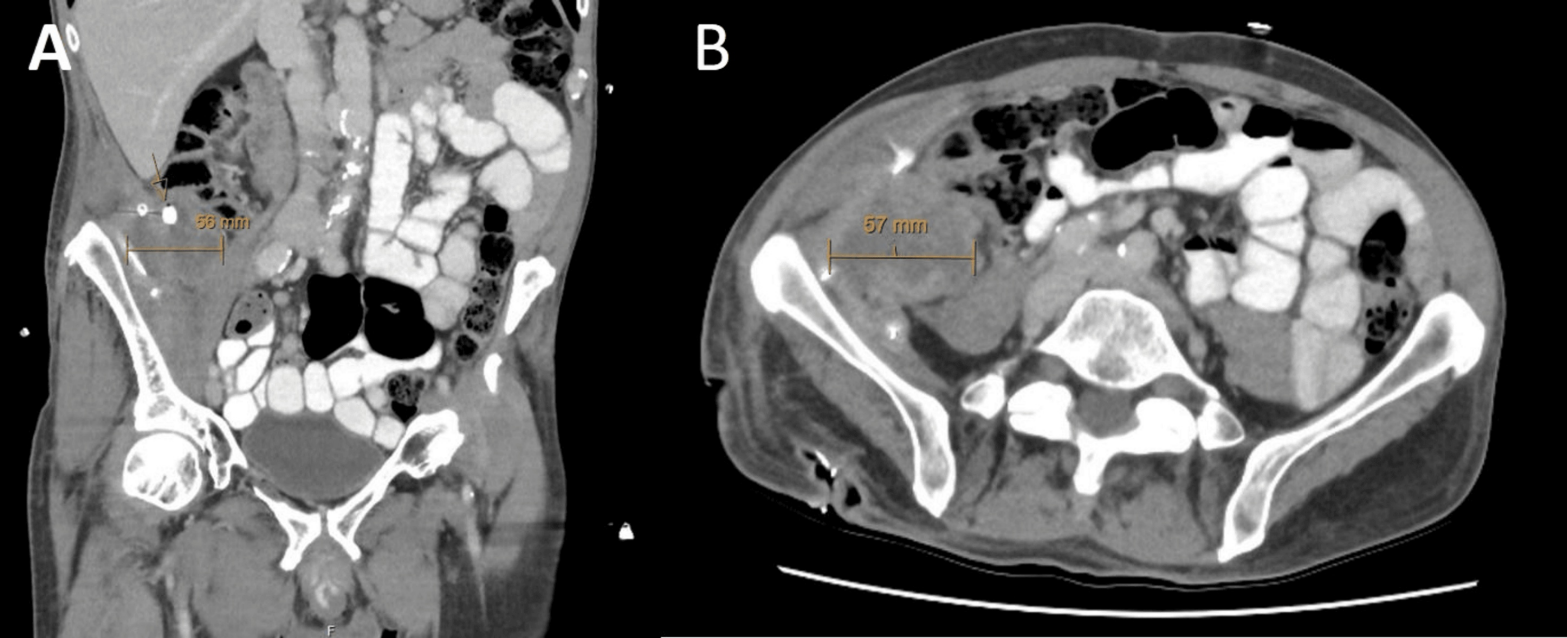
CT of the abdomen and pelvis. (A) Coronal and (B) axial portal venous CT of the abdomen and pelvis demonstrating a persistent intra-abdominal collection with an associated fecalith (arrow) following the removal of the drain.

Seven days following discharge, the patient was reviewed in the outpatient clinic. Output from the deep collection drain remained high at 90-100 mL/day. Wound culture returned positive for *Escherichia coli (E. coli)*, with resistance to amoxicillin/clavulanate but sensitivity to sulfamethoxazole/trimethoprim. A decision was made to commence sulfamethoxazole/trimethoprim twice daily for seven days, with a plan for review one week following to reassess clinical progress and drain output. At the next appointment, the drain outputs had decreased to between 20 and 40 mL per day. Repeat imaging was organized at this point to assess for resolution of this collection, which revealed improvement in the drain-containing superficial portion of this collection and an increase in the intra-abdominal collection surrounding an appendicolith measuring 38 x 56 x 39 mm (Figure 4).

The patient was subsequently readmitted to the hospital for further intravenous antibiotics, amoxicillin-clavulanate 2.2 g three times daily, and preoperative optimization with a high-protein, high-energy diet. At the time of readmission, there was ongoing significant purulent output from the drain of approximately 60 mL per day. He remained systemically well, with some mild ongoing abdominal and back pain. Clinically, the superficial fluctuance and erythema around his drain sites had improved.

**Figure 5:**
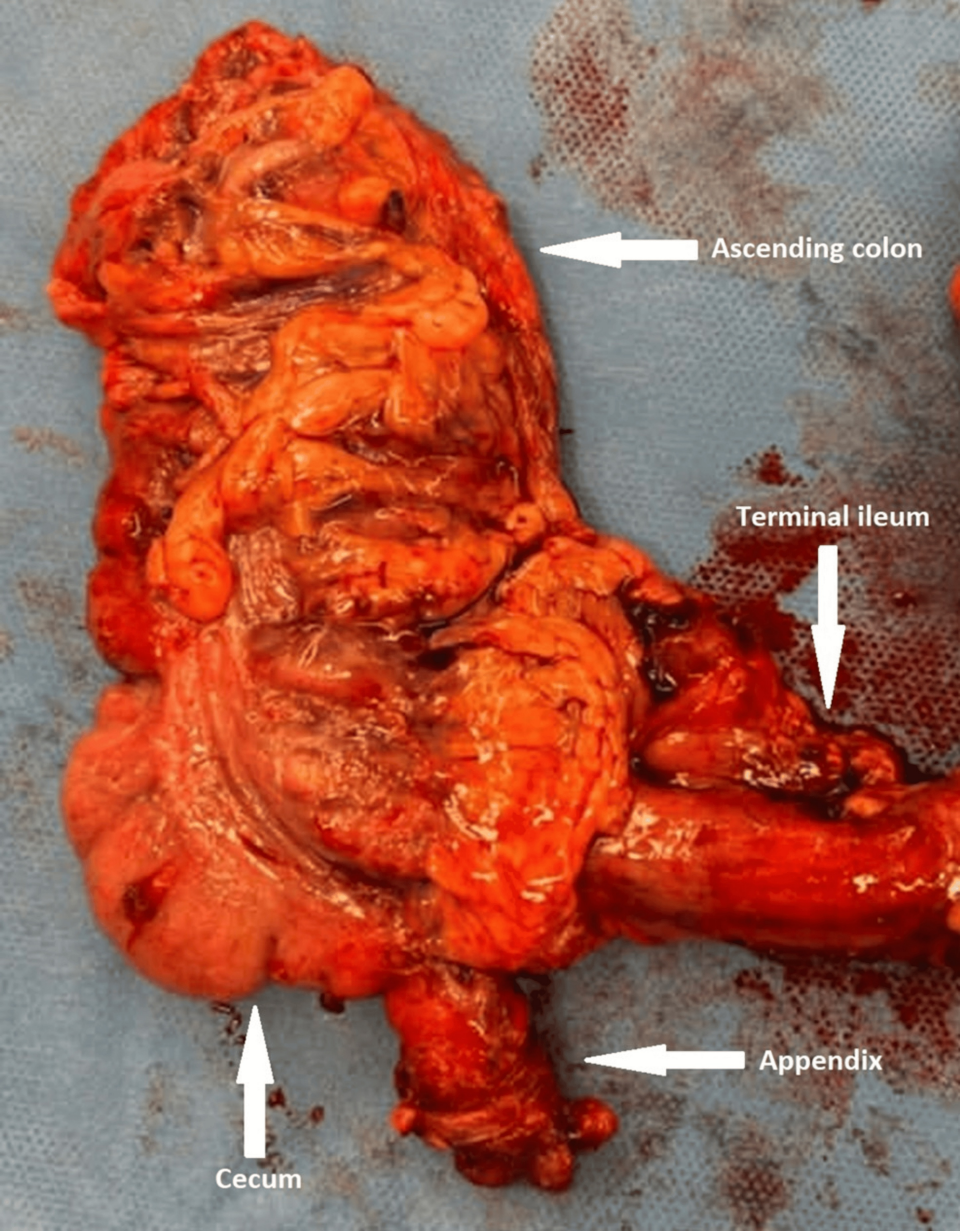
Right hemicolectomy specimen demonstrating dilated appendix inferiorly, with obliteration of the appendiceal tip.

Approximately one month following the initial incision and drainage, a decision was made to pursue operative management with a diagnostic laparoscopy with the goal of sepsis control. At laparoscopy, a large abscess cavity was identified involving the right abdominal wall, densely adherent to the cecum and ascending colon, necessitating laparoscopic right hemicolectomy rather than appendicectomy alone (Figure 5). The appendiceal tip was obliterated within this abscess cavity, consistent with perforated appendicitis. A single fecalith was appreciable within this cavity. A laparoscopic right hemicolectomy was completed with primary side-to-side functional end-to-end anastomosis, without stoma formation. A drainage tube was inserted through the right abdominal wall and pelvis, with the previous flank drains removed. He was admitted to the ward for further antibiotics, analgesia, and titration of diet. His postoperative course was complicated by an ileus. On postoperative day seven, the patient was discharged with resolution of his ileus, with a drain in situ and a further five days of oral amoxicillin-clavulanate 875 mg/125 mg twice daily to complete a 12-day course of antibiotics total. He was reviewed in the outpatient clinic one week following, where drain outputs measured 0 mL and his drain was subsequently removed.

**Figure 6:**
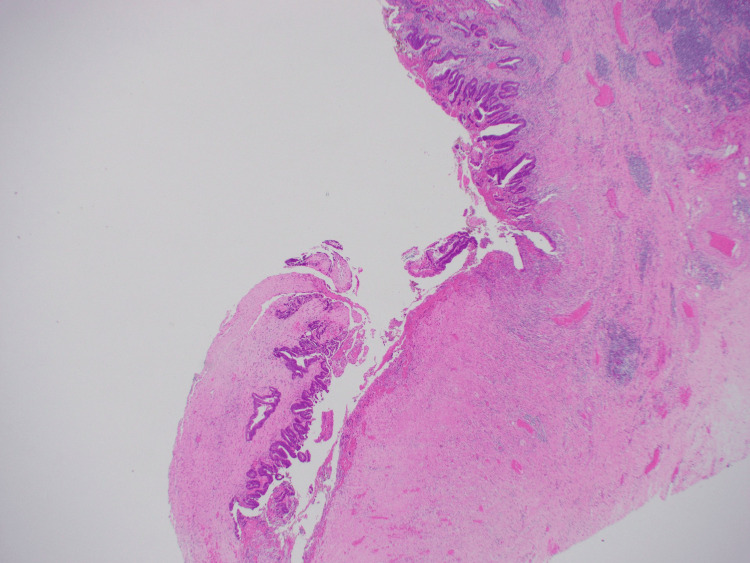
Right hemicolectomy specimen. Demonstrating neoplastic epithelium on the serosal surface of the appendix and within the peri-appendiceal abscess cavity (hematoxylin and eosin (H&E) stain, 20x magnification).

Histopathology of the right hemicolectomy specimen revealed a perforated appendix, with peri-appendiceal abscess formation (Figure 6). Within the appendiceal tip, there was evidence of high-grade adenomatous dysplasia, with dysplastic epithelium lining the abscess cavity. Within this cavity, a focus was suspicious for stromal invasion and equivocal for invasive adenocarcinoma. Twenty-two lymph nodes were retrieved without evidence of metastatic carcinoma. Given the complex pathology, this was referred for a second opinion, with a consensus that this result was consistent with perforated appendicitis with an associated high-grade dysplastic polyp, without evidence of invasive malignancy. His case was discussed at the multidisciplinary meeting, and the patient was referred for a colonoscopy for further evaluation.

**Figure 7:**
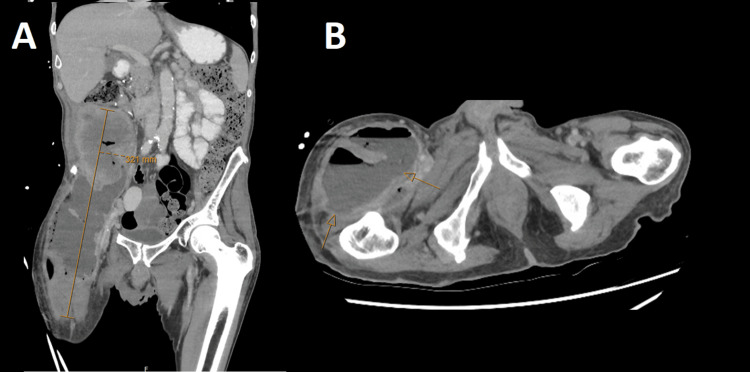
CT of the abdomen and pelvis. (A) Coronal and (B) axial portal venous phase CT of the abdomen and pelvis demonstrating a recurrent intra-abdominal collection with extension to the right mid-thigh (arrows).

Two months following discharge, and prior to colonoscopy, the patient re-presented to the hospital complaining of increased fatigue and lethargy, alongside new right thigh pain, and increasing purulent discharge from his flank wounds. Repeat CT abdomen, and pelvis revealed significant progression of the abdominal collection, now extending to the right groin and anterior thigh with invasion into the proximal anterior thigh muscles, measuring 308 mm in craniocaudal extent (Figure 7). Persistent right flank collection was noted, with invasion into the right psoas muscle.

**Figure 8:**
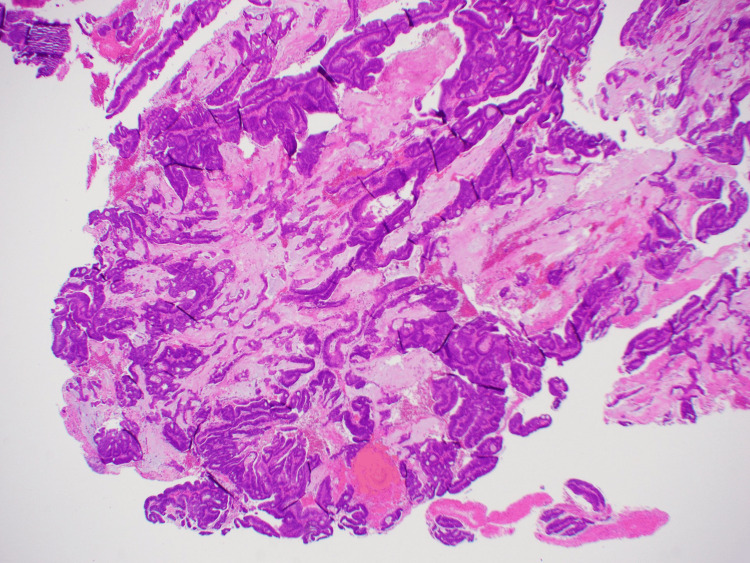
Right hip specimen. Demonstrating strips of neoplastic epithelium within extracellular mucin, in keeping with mucinous adenocarcinoma (hematoxylin and eosin (H&E) stain, 20x magnification).

**Figure 9:**
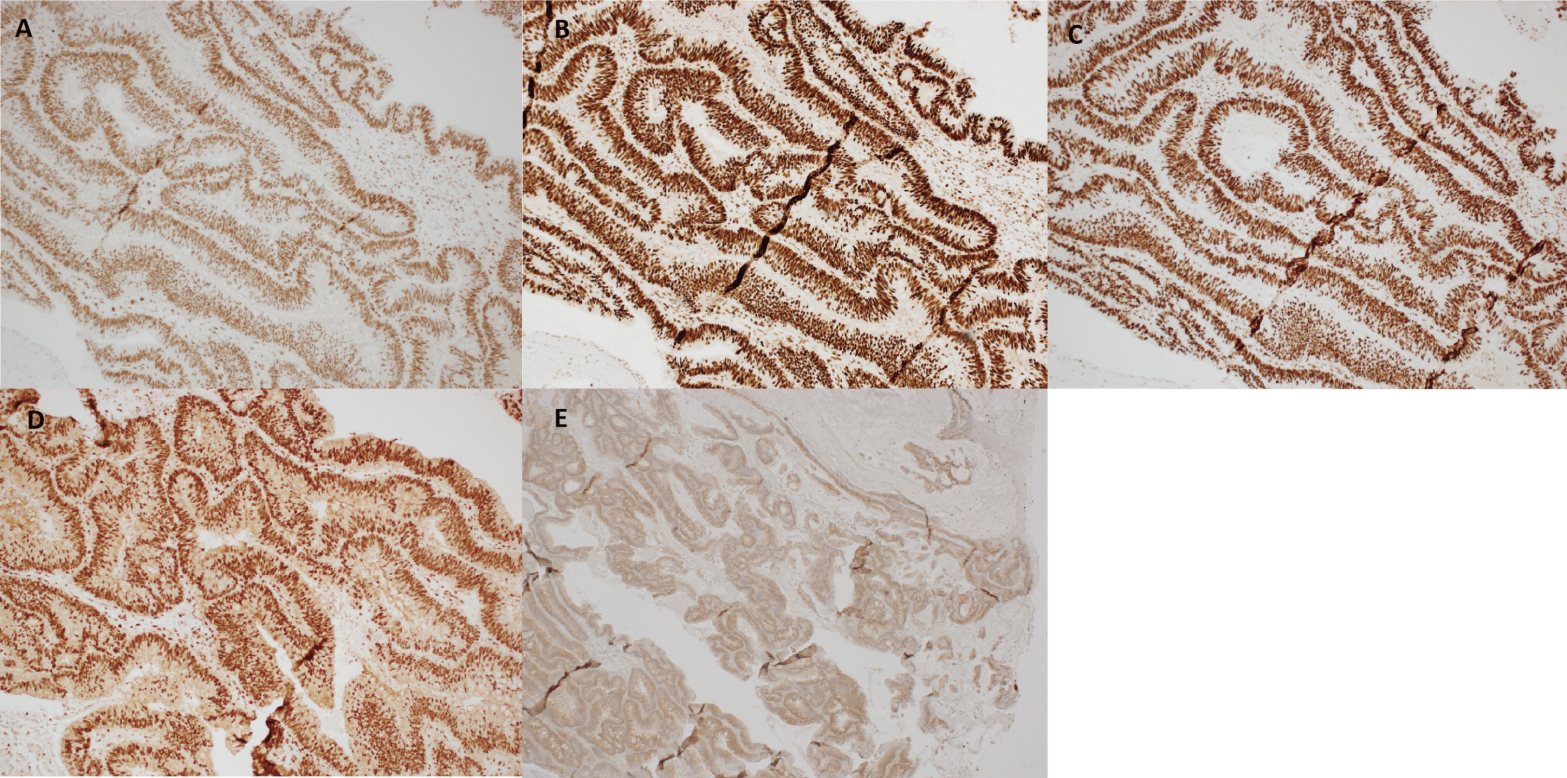
Immunohistochemistry (IHC) of right hip specimen. (A-D) Demonstrating no loss of staining within the mismatch repair proteins: (A) MLH1, (B) MSH2, (C) MSH6, (D) PMS2 (100x magnification). (E) IHC of right hip specimen demonstrating positive BRAF expression (40x magnification).

Re-operation was undertaken, in which the discharging flank sinuses were explored and debrided, with sinus tract and abscess tissue sent for histopathology. Histopathology of this sinus tissue demonstrated psoriasiform hyperplastic and dermal fibrosis; however, tissue from within the abscess confirmed a diagnosis of mucinous adenocarcinoma alongside numerous fragments of high-grade tubulovillous adenoma (Figure 8). Immunohistochemistry revealed no loss of staining within MMR gene proteins (Figure 9). BRAF mutation was detected on immunohistochemistry (Figure 9). Upon discussion with the patient, a decision was made to pursue comfort care without any further active management of this malignancy.

## Discussion

This case illustrates a rare presentation of appendiceal mucinous adenocarcinoma masquerading as perforated acute appendicitis with flank and subsequently thigh abscess formation. Acute appendicitis is a common intra-abdominal pathology, with features of complication including perforation, abscess, and phlegmon formation occurring in 2-7% of patients [11]. In the presence of complications, the risk of associated malignancy significantly increases, with Cerame describing adenocarcinoma of the appendix resulting in perforation in approximately 55% of cases in a 25-year cohort review [12]. Presentation with a fulminant skin and soft tissue infection, including flank and thigh abscess, represents a rare manifestation of perforated appendiceal mucinous adenocarcinoma, with few reported cases in the literature [6-10].

Mir and Singh in 2013 describe a case of appendiceal adenocarcinoma presenting as a flank fistula, with imaging demonstrating a loculated fluid collection within the adjacent abdominal wall [6]. Similarly, Nakao et al. describe a case of a 75-year-old female with a right flank fistula related to appendiceal mucinous cystadenocarcinoma, managed successfully with right hemicolectomy and en bloc tumor resection [7].

Retroperitoneal perforation and subsequent psoas and iliacus invasion from mucinous appendiceal malignancy are well described in the literature [13,14], with tracking within the retroperitoneum allowing disease to spread to the lower limb [15]. Presentation of appendiceal neoplasm with soft tissue infection of the lower limb remains exceedingly rare, with two reported cases in the literature [8,9]. Petrovic et al. describe a 65-year-old woman presenting with a painful right thigh with mild abdominal pain, with an initial diagnosis of perforated appendicitis with psoas abscess formation [8]. This patient was managed with standard appendicectomy and washout of the retroperitoneal and thigh collections, with histopathology demonstrating well-differentiated colonic-type adenocarcinoma of the appendix [8]. There was no evidence of tumor recurrence on follow-up CT 12 months following presentation [8]. Osácar et al. describe a case of a 65-year-old female presenting with right lower quadrant abdominal pain, hyporexia, and weight loss, in which CT demonstrated a pericecal abscess extending to the rectus femoris muscle [9]. This was similarly managed initially with percutaneous measures and incision and drainage, and ultimately required right hemicolectomy for management [9].

Appendiceal malignancies are rare, accounting for approximately 0.5-1% of gastrointestinal tumors, with mucinous adenocarcinoma comprising a minority of cases. Preoperative diagnosis can be challenging, with presentation often mimicking that of acute appendicitis [3,4]. In this reported case, imaging findings and the presence of a fecalith both radiographically and intraoperatively favored a diagnosis of fulminant perforated appendicitis. This diagnosis was confounded by the initial hemicolectomy pathology, which, though noting adenomatous changes with high-grade dysplasia, did not demonstrate evidence of invasive carcinoma despite specialist pathologist input. This highlights the potential for sampling error in the context of perforation and expulsion of dysplastic tissue.

Immunohistochemistry demonstrated positive BRAF expression in this case. BRAF mutations have been described in a subset of appendiceal mucinous neoplasms and are believed to contribute to tumor formation through activation of the MAPK signaling pathway [16]. Although the prognostic significance of BRAF expression in appendiceal mucinous adenocarcinoma remains uncertain, this appears to represent a distinct molecular subtype compared with colorectal cancer, with potential responsiveness to BRAF-targeted therapy [16]. Given the patient's decision to pursue a comfort care approach, further discussion regarding the utility of BRAF-targeted therapy was not undertaken in this case.

In this case, the presence of progressive and recurrent abscesses despite appropriate operative intervention raised suspicion for underlying malignancy. Persistent or non-resolving abscesses have been described in association with perforated mucinous adenocarcinoma of the appendix and should prompt reconsideration of the diagnosis [17,18]. Moreover, care should be taken to manage those patients with non-operatively managed perforation, with consideration of colonoscopy, contrast-enhanced CT, and staged appendicectomy, particularly within adult populations, to ensure underlying malignancy is not missed [19].

Management was further complicated by the rural setting, where access to interventional radiology was limited. In the contemporary setting, image-guided percutaneous drainage is commonly utilized for the management of intra-abdominal or retroperitoneal collections. In the absence of this resource, surgical incision and drainage were required to achieve initial control of sepsis; recurrent procedures were necessary for adequate control of persistent collections. This underscores the challenges inherent in managing complex intra-abdominal sepsis in resource-constrained environments.

## Conclusions

Perforated appendiceal mucinous adenocarcinoma may present rarely as a flank and thigh abscess, related to retroperitoneal perforation with tracking along fascial planes. In adults presenting with complicated appendicitis, particularly with persistent or recurrent collections, a high index of suspicion is required to rule out underlying malignancy. This case highlights the diagnostic challenge associated with an atypical presentation and underscores the importance of clinical vigilance and appropriate patient follow-up and imaging in these scenarios.
